# Bromide of Ammonium in Dysmenorrhœa

**Published:** 1868-10

**Authors:** A. A. Dunn

**Affiliations:** Chicago, Ill.


ARTICLE XLI.

### BROMIDE OF AMMONIUM IN DYSMENORRHŒA.

By A. A. Dunn, M.D., Chicago, Ill.

June 10th, 1867, an unmarried lady, aged 32 years, consulted me about her painful monthly periods. She is short, quite plump, without much of the adipose, bowels regular, general health good, cheeks ruddy.

Her mother states that, from her earliest menstrual period down to the date just given, the young lady has had but few occasions of comparative comfort during her "turns." Pain in great severity behind the pubes, in the groins, in the lumbar and sacral regions, in the right leg, in the head, accompanied by flushed face and frequently with febrile movement, ensued nearly always at each return of her courses, which were very regular as to time of return, duration, quality and quantity of flow. The discomfort would commence with the ovarian and uterine engorgement, reach its maximum in from 36 to 60

hours, subsiding after the appearance of the catamenia in full flow.

No attempt at radical relief was made during those long years, so far as I could learn. Occasionally, a physician was called in to afford relief temporarily, during a more than usually severe paroxysm of pain.

She consulted me just after the cessation of an ovarian evolution. I prescribed am. brom. ℥ss., aquæ ℥iv., a tablespoonful of which was to be taken in half tumbler of water three times a day. At the return of the menstrual period, if the bowels were at all inactive, directed an effervescing purgative to be taken. If, as usual, pain was established, she was to inject into the vagina a gallon or two of water, more than blood warm, three or four times a day. As she was a good liver, I abated somewhat the more stimulating articles of diet in which she was prone to indulge, but by no means restricted her to *low diet;* and what restriction I did place upon her, was more particularly directed to the period a week prior to and during the catamenial effort.

The next ensuing period was passed in almost absolute comfort; and so of the second, third, and fourth, when the quantity of medicine was reduced one-half. The fifth period passing with equal comfort, the medicine was suspended, and the sixth period came and went with as little discomfort to the patient as any of the previous five. Such continued to be the case down to last period, recently passed, when, after partaking freely of ice-cream, and, immediately after, bathing in the ocean, the catamenial flow came on, anticipating the usual period four days, and was accompanied by no inconsiderable amount of distress, though by no means so violent as was usual prior to the exhibition of the bromide. Neither the injection nor purgative was resorted to on a single occasion, as the requirements did not exist. The medicine affected the appetite unfavorably not at all, so far as I have been able to learn, and certainly did not during the three months she was immediately under my eye. The urine was not subjected to chemical analysis, and I may say here, she has never complained of ardor urinæ before or since taking the bromide. The pulse maintained its usual force and frequency while the patient was where I could observe her condition.

In this case there was evident benefit derived from the use of the medicine, or, there was a strikingly marked coincidence between the commencing and progressive improvement of the patient, and the use of the compound. The results in this case cannot be considered conclusive as to any effect whatever of the medicine exhibited; but, placed in the category of the results following its exhibition in similar cases, they may contribute somewhat towards reaching sound conclusions as to the therapeutic effect to be expected from the use of the drug.

Sedation is the effect claimed for the article under consideration. My observations have not led me to attribute to it any such effect upon the circulation. If we must consider it a nervous sedative, then it belongs to that class of medicines known as anodynes, or to that other class which allays perturbation so common to hysterical conditions, and which are denominated nervous stimulants. Among possible things, the drug may have a *peculiar* effect upon the nerve-tissue, not now understood, differing from that of our anodynes proper, and from the list of nervous stimulants. Neither my experience nor my reading warrants a positive opinion on this point.

